# Diffuse large B cell lymphoma of thyroid as a masquerader of anaplastic carcinoma of thyroid, diagnosed by FNA: a case report

**DOI:** 10.1186/1742-6413-3-23

**Published:** 2006-10-19

**Authors:** Yahya Daneshbod, Shapour Omidvari, Khosrow Daneshbod, Shahrzad Negahban, Mehdi Dehghani

**Affiliations:** 1 Department of Cytopathology, Dr. Daneshbod Pathology Laboratory, Shiraz, Iran; 2 Department of Hematopathology, Dr. Daneshbod Pathology Laboratory, Shiraz, Iran; 3 Department of Pathology, Dr. Daneshbod Pathology Laboratory, Shiraz, Iran; 4 Department of Radiation-Oncology, Nemazee Hospital, Shiraz University of Medical Sciences, Shiraz, Iran; 5 Department of Hematology-Oncology, Nemazee Hospital, Shiraz University of Medical Sciences, Shiraz, Iran

## Abstract

**Background:**

Both thyroid lymphoma and anaplastic carcinoma of thyroid present with rapidly growing mass in eldery patients. Anaplastic carcinoma has high mortality rate and combination of surgery, radiation therapy and multidrug chemotherapy are the best chance for cure. Prognosis of thyroid lymphoma is excellent and chemotherapy for widespred lymphoms and radiotherapy with or without adjuvant chemotherapy for tumors localized to the gland, are the treatment of choice.

**Case report:**

This article reports a 70 year old man presenting with diffuse neck swelling and hoarseness of few weeks duration. Fine needle aspiration was done and reported as anaplastic carcinoma of thyroid which thyroidectomy was planned. The slides were sent for second opinion. After review, with initial diagnosis of anaplastic carcinoma versus lymphoma, immunocytochemical study was performed. Smears were positive for B cell markers and negative for cytokeratin, so with the impression of diffuse large B cell lymphoma, the patient received two courses of chemotherapy by which the tumor disappeared during two weaks.

**Conclusion:**

Despite previous reports, stating easy diagnosis of high-grade thyroid lymphoma on the grounds of cytomorphological features we like to emphasize, overlapping cytologic features of the curable high grade thyroid lymphoma form noncurable anaplastic thyroid carcinoma and usefulness of immunocytochemistry to differentiate these two disease.

## Case presentation

**Figure 1:**
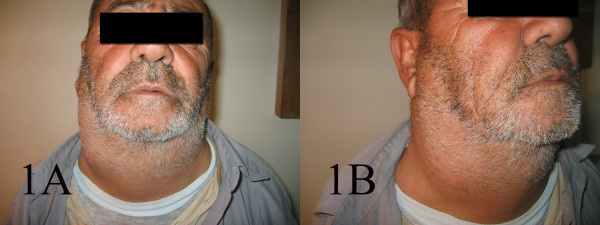
Diffuse neck swelling.

**Figure 2:**
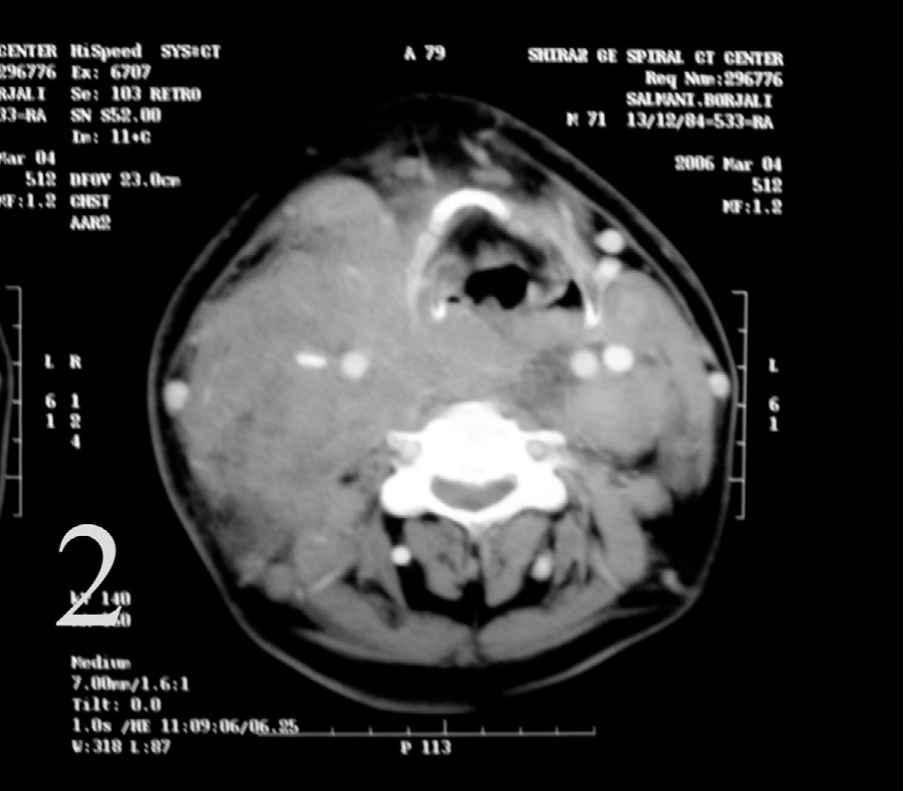
CT scan was performed which showed a large cervical mass arising form thyroid and extending to left cervical soft tissue with displacement of trachea.

**Figure 3:**
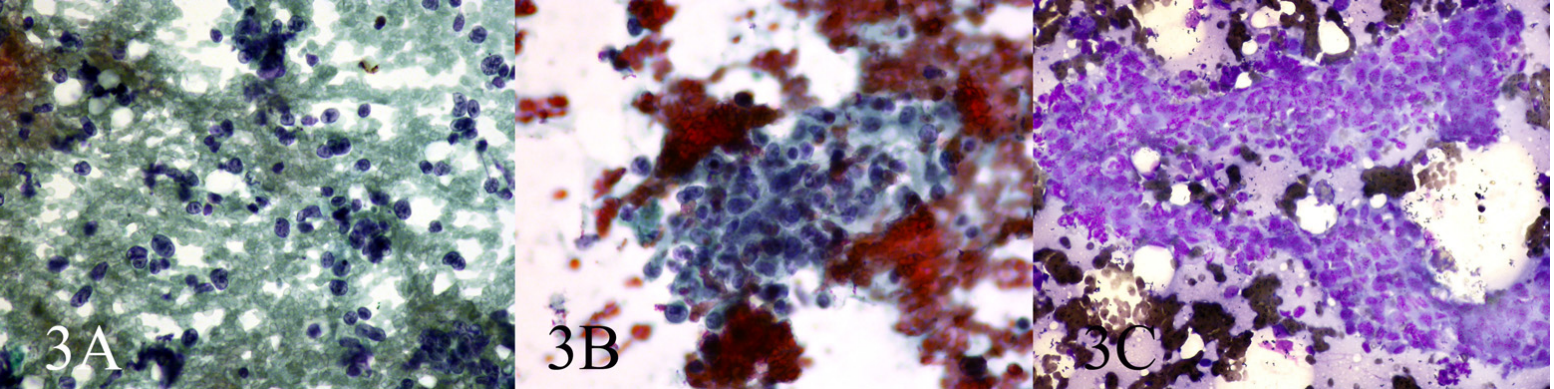
Smears were cellular, consisting of isolated and clusters of pleomorphic malignant cells with irregular nuclear membrane, prominent nucleoli (Papanicolaou, Papanicolaou, Wright, 200, 200, 200).

**Figure 4:**
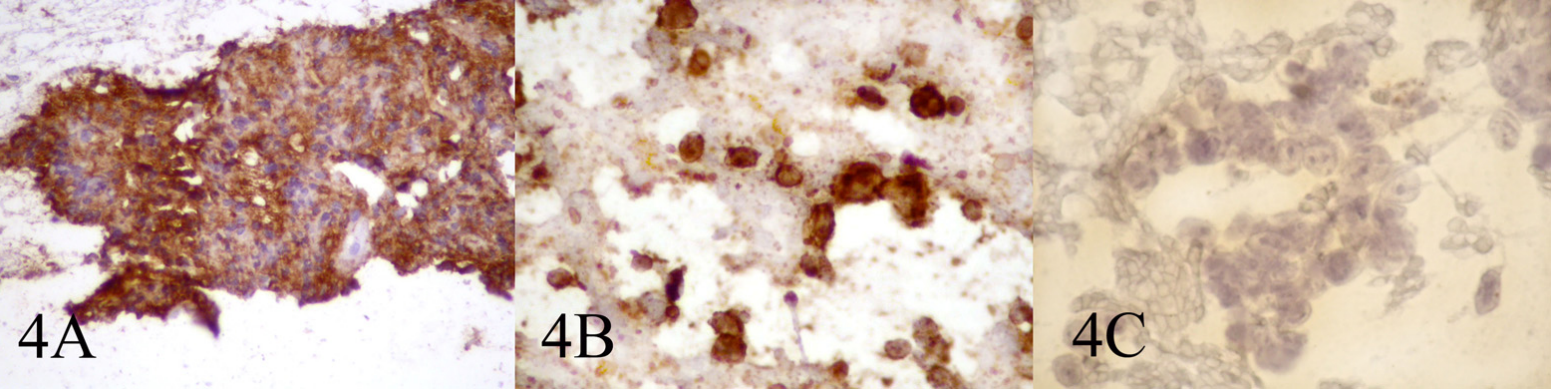
Immunocytochemical stain for LCA (A) CD20 (B), and CK (C).

**Figure 5:**
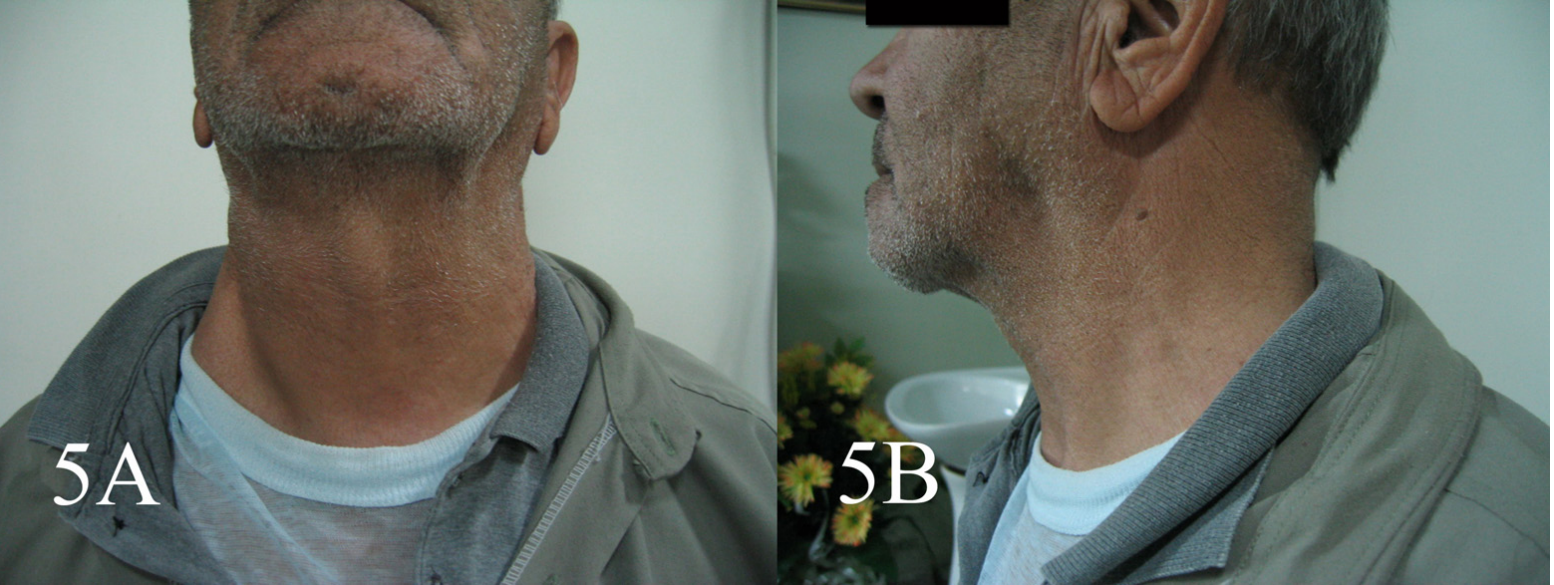
Complete resolution of neck swelling one week after receiving chemotherapy.

A 70 year old farmer presented with diffuse neck swelling and hoarseness of one month duration (Fig 1A and B). CT scan was performed which showed a large cervical mass arising form thyroid and extending to right cervical soft tissue with displacement of trachea (Fig 2). FNA was done and reported as anaplastic carcinoma of thyroid. Thyroidectomy with debulking of the tumor was planned for the patient. The FNA smears were referred to us for a second opinion. Smears were cellular, consistening of isolated and clusters of pleomorphic malignant cells with irregular nuclear membrane, prominent nucleoli (Fig 3A, B andC). With the initial cytologic diagnosis of anaplastic carcinoma versus lymphoma, immunocytochemical staining on prefixed smears with a panel of antisera including cytokeratin, EMA, LCA, CD20, CD3, and immunoglobulin light chains(all Zymed antisera) were performed. Smears were positive for LCA, CD20 (Fig. 4A and B), λ light chain and negative for cytokeratin (Fig 4C). With the impression and diffuse large B-cell lymphoma, the patient received two courses of chemotherapy with complete resolution of his lesion one week later (Fig 5A and B).

## Conclusion

Cytologic specimens from malignant diffuse large B cell lymphomas are characterized by an almost monotonous population of noncohesive atypical cells. In most cases, these cells are large, with irregular vesicular nuclei and prominent nucleoli. Cytologic features of anaplastic carcinoma are pleomorphic, round, oval or spindle shaped cells either isolated or in tissue fragments. Although lack of cohesion and absence of lymphoglandular body are said to be against anaplastic carcinoma [1-6], however thyroid lymphoma can also show pleomorphism with mostly cells that are dispersed or arranged into lymphoid tissue fragments indistinguishable from anaplastic carcinoma.

Lymphoma, particularly non Hodgkin's B-cell lymphomas accounts for 1–3% of primary thyroid malignancies and most commonly arises in the setting of Hashimoto's thyroiditis. Diffuse large B-cell lymphomas and extranodal marginal zone lymphomas of MALT type account for the majority of cases [7-9].

Both thyroid lymphoma and anaplastic carcinoma of thyroid present in elderly patients with rapidly growing mass and can lead to symptoms of tracheal or laryngeal compression. Extra thyroid extension is encountered at the time of initial presentation in most cases. The mortality rate in anaplastic carcinoma is over 95% and the mean survival is less than 6 months. Currently, the best chance for cure are obtained with a combinations of surgery, radiation therapy and multidrug chemotherapy. However treatment of widespread thyroid lymphoma is chemotherapy and if the tumor is localized to the gland only or the regional lymph nodes, radiation therapy with or without adjuvant chemotherapy appears warranted. The prognosis of thyroid lymphoma is excellent [9-11].

Despite previous reports, stating easy diagnosis of high-grade thyroid lymphoma on the grounds of cytomorphological features [1-6], we like to emphasize, overlapping cytologic features of the curable high grade thyroid lymphoma form noncurable anaplastic thyroid carcinoma and usefulness of immunocytochemistry to differentiated these two disease.
